# Correction: Allocation of the household food budget among shopping basket items: How is it influenced by promotions?

**DOI:** 10.1371/journal.pone.0322910

**Published:** 2025-04-22

**Authors:** The PLOS One Staff

## Notice of Republication

This article was republished on March 7, 2025, to correct an error in the XML article and PDF. There was an error in the placement of some text after Table 4 that was introduced during type setting. The publisher apologizes for this error.
