# Supplementary material for: Allocation of the household food budget among shopping basket items: How is it influenced by promotions?
Source: PLoS One. 2024 Jun 5;19(6):e0304911. doi: 10.1371/journal.pone.0304911 (PMC11152314; doi:10.1371/journal.pone.0304911)
Supplement: S1 Appendix — (DOCX) [file pone.0304911.s001.docx]

**Supplementary Appendix**

**Data preparation**

The categories’ prices and the promotions’ indices were computed following those in Drèze et al. (42), using the weighted average of the prices paid by the households for each product in each food category in each shopping trip as follows;

Price per category:

$P_{gt}^{(h)}=\sum_{s=1}^{s} p_{st}\cdot w_{s}^{(h)}$ (12)

Promotion index per category:

${Pm}_{gt}^{(h)}=\sum_{s=1}^{s} {pm}_{st}\cdot w_{s}^{(h)}$ (13)

Expenditure per category:

$E_{gt}^{h}=\sum_{s=1}^{s} p_{st}\cdot q_{st}^{(h)}$ (14)

Where:

$w_{s}^{(h)}=\frac{\sum_{t=1}^{T} p_{st}\cdot q_{st}^{(h)}}{\sum_{t=1}^{T} \sum_{s=1}^{S} p_{st}\cdot q_{st}^{(h)}}$ (15)

*q_st_* : Quantity of the product *(s)* during shopping trip*(t)*.

*p_st_* : Price of product *(s)* during shopping trip*(t)*.

*pm_st_* : A dummy variable that takes a value of 1 if a promotion was used and 0 otherwise.

*(T)*: Number of shopping trips made by the household.

*(s)*: Number of products in each food category *(g)*.

**The probit model (Heien and Wessells, 1990) Approach**

The first stage of the censored demand system, is estimated by a probit regression model, that calculates the probability that a household will consume or decide to purchase a good during his shopping trip. According to Heien and Wessells (50), the model is presented as follows;

|  | $w_{hgt}^{*}=f\left( X_{ht}, Z_{n},p_{hgt} \right)$ | (16) |
| --- | --- | --- |

Where $w_{hit}^{*}$ takes a value of 1 if a household consumes the food category during period $t$ ($w_{hit}>0)$ and 0 otherwise, $X_{ht}$ represents the total real expenditure, $Z_{n}$ is a vector of a sociodemographic variables, $p_{hgt}$ is the price of the category *g* paid by the household *h* during shopping trip *t.*

The regression is then used for each household to compute an Inverse Mills Ratio (IMR), which is equal to the [ratio](https://en.wikipedia.org/wiki/Ratio) of the [probability density function](https://en.wikipedia.org/wiki/Probability_density_function) to the [cumulative distribution function](https://en.wikipedia.org/wiki/Complementary_cumulative_distribution_function) of a distribution. When the household consumes, the IMR is,

$R_{hit}=\frac{\phi(X_{h} , Z_{h},p_{hgt})}{\Phi(X_{h}, Z_{h,}p_{hgt})}$ otherwise, $R_{hit}=\frac{\phi(X_{h}, Z_{h},p_{hgt})}{(1-\Phi\left( X_{h}, Z_{h},p_{hgt} \right)}$

The IMR is then used as an instrument to explain the censored nature of the dependent variable in the second stage of demand system estimation.

**Supplementary Tables**

**Table S1. Categories’ composition and summary statistics**

| Food categories | Category composition | Number of products | Level of censoring (%) |
| --- | --- | --- | --- |
| Grains and grain-based products | -Fresh bread  -Frozen bread  -Burger and hotdog breads  -Flour and semolina  -breakfast cereals | 12 | 46.94 |
| Vegetables and vegetable products | -Canned vegetables  -Dehydrated vegetables  -Fresh vegetables  -Prepared salads  -Frozen vegetables | 15 | 59.84 |
| Starchy roots, tubers Legumes, nuts and oilseeds | - Dehydrated mashed potato  -Pulses  -Nuts | 6 | 76.18 |
| Fruit, fruit products and fruit and vegetable juices | -Fresh fruits  -Canned fruits  -Compotes  -Fruits in syrup  -Plant-based drinks | 8 | 47.44 |
| Meat | -Beef  -Pork  -Chicken  -Rabbit  -Processed meats  -Other meats | 40 | 48.06 |
| Fish and other seafood | -Canned Fish and seafood  -Smoked fish  -Fresh fish and seafood | 20 | 57.01 |
| Milk, dairy products and milk product imitates | -Milkshakes  -Yogurts  -Milk powder  -Milk | 8 | 46.63 |
| Cheese | -All types of cheese | 15 | 58.22 |
| Sugar and confectionery and prepared desserts | -Jams  -Honey  -Cakes and pastries  -Ice cream  -Chocolate  -Fresh desserts and bakery  -Other sweets | 35 | 43.43 |
| Composite dishes (animal and vegetable) | -frozen ready meals  -Concentrated soups  - Soups and creams  - Frozen pizzas and ready to eat pizzas  -Rice and pasta meals  -Fresh cooked dishes | 18 | 59.34 |
| Snacks and other food | -Salted snacks  -Crackers  -Pickles  -Crisps  -Popcorn  -Appetizers | 8 | 73.29 |
| Drinks | -Sodas and carbonated soft drinks  -Water  -Beer  -Wine  -Juices | 63 | 39.67 |
| Residual category | -Coffee  -Tea  -Cooking oils  -Other frozen products  -Nutritional bars  -Whipped cream | 53 | 49.92 |

**Table S2. Probit model results**

| **Variables** | **Grains** | **Vegetables** | **Starchy** | **Fruits** | **Meat** | **Fish** | **Milk** | **Cheese** | **Sugar** | **Composite** | **Snacks** | **Drinks** | **Residual** |
| --- | --- | --- | --- | --- | --- | --- | --- | --- | --- | --- | --- | --- | --- |
| Age | 0.004  (0.004) | 0.0004 (0.004) | 0.007 (0.004) | 0.007* (0.004) | -0.007* (0.004) | -0.013***  (0.004) | 0.007* (0.004) | -0.004  (0.004) | 0.015*** (0.004) | -0.004 (0.004) | -0.017*** (0.004) | 0.014*** (0.004) | 0.010*** (0.004) |
| ***Social class*** |  | | | | | | | | | | | | |
| Middle class | -0.066  (0.065) | 0.144** (0.069) | 0.167** (0.078) | -0.030 (0.066) | 0.216*** (0.069) | 0.225*** (0.070) | -0.168** (0.073) | -0.111 (0.068) | 0.060 (0.067) | -0.225*** (0.068) | 0.054 (0.080) | -0.081 (0.071) | 0.205*** (0.069) |
| Middle-high class | -0.086  (0.073) | 0.186** (0.076) | 0.045 (0.086) | -0.007 (0.074) | 0.118 (0.077) | 0.270*** (0.078) | -0.198*** (0.075) | 0.016 (0.075) | 0.093 (0.074) | -0.123 (0.075) | 0.112 (0.088) | -0.184** (0.079) | 0.120 (0.077) |
| Middle-low class | -0.424***  (0.077) | 0.016 (0.081) | 0.221** (0.091) | -0.115 (0.078) | 0.292*** (0.081) | 0.233*** (0.082) | -0.385*** (0.079) | -0.049 (0.081) | 0.023 (0.079) | -0.404*** (0.081) | 0.037 (0.098) | 0.156* (0.084) | 0.138* (0.081) |
| Immigrant | -0.083**  (0.052) | 0.034 (0.054) | -0.092  (0.060) | 0.010  (0.053) | 0.031  (0.055) | -0.142** (0.056) | -0.119** (0.054) | 0.187*** (0.054) | -0.096* (0.054) | -0.086 (0.054) | 0.010 (0.063) | -0.022 (0.057) | -0.010  (0.055) |
| Expenditures | 0.010***  (0.000) | 0.015*** (0.001) | -0.013*** (0.001) | 0.014*** (0.001) | 0.021*** (0.001) | 0.019*** (0.001) | 0.015*** (0.001) | 0.014*** (0.001) | 0.013*** (0.001) | 0.015*** (0.001) | 0.009*** (0.001) | 0.022*** (0.001) | 0.019*** (0.001) |
| ***Life cycle*** |  | | | | | | | | | | | | |
| Single-parent households | 0.358**  (0.157) | 0.435*** (0.158) | 0.338* (0.178) | -0.113 (0.150) | 0.389** (0.160) | 0.176 (0.166) | 0.851*** (0.163) | 0.686*** (0.168) | 0.465*** (0.151) | 0.859*** (0.177) | 0.929*** (0.216) | 0.201 (0.158) | -0.091 (0.156) |
| Independent Youth | 0.196  (0.239) | 0.527** (0.246) | 0.237 (0.281) | 0.234 (0.241) | 0.593** (0.256) | 0.821*** (0.258) | 0.496*** (0.249) | 0.494* (0.254) | 0.738*** (0.246) | 1.024*** (0.259) | -0.510 (0.350) | -0.170 (0.251) | 0.031 (0.248) |
| Adult Couples without children | 0.496***  (0.155) | 0.045 (0.155) | -0.032 (0.178) | -0.264* (0.147) | 0.322** (0.156) | 0.423*** (0.162) | 0.579*** (0.159) | 0.503*** (0.166) | 0.089 (0.147) | 0.577*** (0.175) | 0.416* (0.216) | -0.158  (0.153) | -0.295* (0.153) |
| Couples with middle-aged children | 0.314**  (0.152) | -0.021 (0.154) | 0.276 (0.174) | 0.031 (0.146) | 0.277* (0.155) | 0.147 (0.161) | 0.723*** (0.158) | 0.359** (0.164) | 0.327** (0.146) | 0.686*** (0.173) | 0.778*** (0.212) | 0.069 (0.152) | -0.257* (0.151) |
| Couples with adult children | 0.267*  (0.150) | 0.232 (0.157) | 0.007 (0.180) | -0.119 (0.149) | 0.179 (0.159) | 0.288* (0.165) | 0.703*** (0.161) | 0.381** (0.168) | -0.098 (0.149) | 0.710*** (0.176) | 0.535** (0.218) | 0.048 (0.155) | -0.114 (0.155) |
| Couples with small children | 0.524***  (0.159) | 0.003 (0.167) | -0.055 (0.191) | 0.068 (0.158) | 0.316* (0.169) | 0.106 (0.174) | 0.813*** (0.171) | 0.329* (0.177) | 0.398** (0.160) | 0.570***  (0.185) | 0.168 (0.227) | 0.199 (0.168) | -0.133 (0.166) |
| Young couples without children | 0.829***  (0.186) | 0.082 (0.193) | 0.335 (0.217) | -0.003 (0.185) | 0.317 (0.196) | -0.019 (0.201) | 1.017*** (0.196) | 0.305 (0.202) | 0.600*** (0.187) | -0.927*** (0.208) | 0.360 (0.250) | 0.182 (0.195) | 0.288 (0.192) |
| Retired | 0.208  (0.169) | 0.296* (0.177) | -0.268 (0.205) | -0.193 (0.169) | 0.273 (0.179) | 0.823*** (0.185) | 0.453** (0.181) | 0.170 (0.189) | -0.468*** (0.170) | -0.414** (0.196) | -0.137 (0.254) | -0.514*** (0.178) | -0.458*** (0.176) |
| ***Province*** |  | | | | | | | | | | | | |
| Gerona | 0.234***  (0.076) | -0.086 (0.079) | 0.275*** (0.084) | -0.039 (0.076) | -0.044 (0.078) | 0.070 (0.079) | -0.085 (0.076) | 0.195** (0.077) | 0.058 (0.076) | 0.142* (0.077) | -0.153 (0.096) | 0.070 (0.078) | 0.072 (0.078) |
| Lerida | 0.040  (0.091) | -0.028 (0.096) | 0.165 (0.105) | -0.038 (0.093) | -0.348*** (0.098) | 0.110 (0.097) | 0.036 (0.093) | 0.161* (0.096) | 0.064 (0.093) | -0.419*** (0.118) | -0.291** (0.115) | -0.263*** (0.096) | 0.228* (0.095) |
| Tarragona | -0.129  (0.104) | 0.092 (0.108) | -0.248* (0.142) | 0.004 (0.106) | -0.179 (0.111) | 0.207* (0.110) | -0.090 (0.107) | 0.012 (0.109) | -0.229** (0.106) | -0.156 (0.110) | 0.233* (0.120) | 0.018 (0.114) | -0.294*** (0.112) |
| Price | -0.029  (0.025) | -0.027 (0.021) | 0.335*** (0.028) | -0.006 (0.024) | -0.068*** (0.025) | 0.074*** (0.026) | 0.015 (0.029) | 0.295*** (0.029) | -0.020 (0.030) | 0.077*** (0.025) | 1.004*** (0.040) | -0.282*** (0.021) | -0.197*** (0.020) |
| Constant | -0.792***  (0.253) | -1.134*** (0.265) | -1.834*** (0.297) | -0.788*** (0.258) | -0.840*** (0.268) | -0.811*** (0.273) | -1.339*** (0.265) | -1.038*** (0.269) | -1.365*** (0.258) | -1.084*** (0.275) | -0.873*** (0.330) | -1.541*** (0.274) | -1.164*** (0.265) |
| Log likelihood | -2303.007 | -2101.368 | -1632.209 | -2224.847 | -2025.241 | -2006.665 | -2165.573 | -2068.610 | -2196.755 | -2071.003 | -1390.887 | -1904.572 | -2038.717 |
| Pseudo R^2^ | 0.065 | 0.125 | 0.166 | 0.098 | 0.179 | 0.176 | 0.124 | 0.146 | 0.100 | 0.140 | 0.328 | 0.204 | 0.175 |

Source: Own elaboration based on Kantar Worldpanel data.

Notes: standard errors are presented in parenthesis, Sig: Significance level. ***, **, * indicate significance at p <0.001, p < 0.05 and p <0.01, respectively.

**Table S3. Coefficients of the estimated IMR and the socio-demographic variables**

| Variable | Eq 1 | Eq 2 | Eq 3 | Eq 4 | Eq 5 | Eq 6 | Eq 7 | Eq 8 | Eq 9 | Eq 10 | Eq 11 | Eq12 |
| --- | --- | --- | --- | --- | --- | --- | --- | --- | --- | --- | --- | --- |
| IMR | 0.067***  (0.009) | 0.124***  (0.004) | 0.094***  (0.003) | 0.090***  (0.008) | 0.040***  (0.008) | 0.117***  (0.006) | 0.071***  (0.008) | 0.124***  (0.005) | 0.029***  (0.009) | 0.146***  (0.006) | 0.098***  (0.003) | 0.003  (0.009) |
| *Household size* |  | | | | | | | | | | | |
| 2 | 0.007  (0.009) | -0.014**  (0.006) | -0.009***  (0.004) | 0.009  (0.009) | -0.027**  (0.013) | -0.000  (0.011) | -0.011  (0.011) | 0.008  (0.007) | 0.011  (0.012) | 0.012  (0.008) | -0.001  (0.005) | -0.014  (0.015) |
| 3 | 0.006  (0.009) | -0.011*  (0.006) | -0.005  (0.0004) | 0.013  (0.010) | -0.026*  (0.013) | -0.034***  (0.011) | -0.004  (0.011) | -0.002  (0.007) | 0.004  (0.012) | 0.007  (0.009) | 0.002  (0.005) | 0.018  (0.015) |
| 4 | 0.017*  (0.010) | -0.024***  (0.006) | -0.006  (0.004) | -0.006  (0.010) | -0.035**  (0.014) | -0.041***  (0.012) | 0.025**  (0.011) | -0.010  (0.007) | 0.011  (0.012) | -0.003  (0.009) | 0.011**  (0.005) | 0.003  (0.015) |
| +5 | -0.002  (0.012) | -0.019**  (0.008) | -0.009  (0.005) | 0.043***  (0.013) | -0.058***  (0.018) | -0.066***  (0.015) | 0.006  (0.015) | -0.021**  (0.010) | -0.005  (0.015) | 0.003  (0.011) | -0.0006  (0.006) | 0.080***  (0.019) |
| *Social class* |  | | | | | | | | | | | |
| Middle-class | -0.001  (0.006) | 0.005  (0.004) | -0.009***  (0.003) | -0.008  (0.007) | 0.020**  (0.009) | 0.021***  (0.008) | -0.009  (0.008) | -0.017***  (0.005) | -0.024***  (0.008) | -0.025***  (0.006) | 0.002  (0.003) | -0.002  (0.010) |
| Middle-high class | -0.001  (0.007) | 0.010**  (0.004) | -0.009***  (0.003) | 0.0004  (0.007) | 0.011  (0.010) | 0.026***  (0.009) | -0.019**  (0.008) | -0.007  (0.005) | -0.012  (0.009) | -0.022***  (0.006) | -0.0002  (0.004) | -0.005  (0.011) |
| Middle-low class | -0.015**  (0.008) | 0.006  (0.005) | 0.004  (0.003) | -0.013  (0.008) | 0.027**  (0.011) | 0.037***  (0.009) | -0.019**  (0.009) | -0.019***  (0.006) | -0.032***  (0.010) | -0.029***  (0.007) | 0.004  (0.004) | 0.031**  (0.012) |
| *Region* |  | | | | | | | | | | | |
| No metropolitan area | 0.013***  (0.004) | -0.003  (0.003) | -0.004**  (0.002) | -0.0005  (0.005) | -0.014**  (0.006) | 0.013**  (0.005) | 0.001  (0.005) | 0.012***  (0.003) | 0.019***  (0.006) | -0.005  (0.004) | -0.007***  (0.002) | -0.016**  (0.007) |
| *Age* | -0.0004**  (0.000) | -0.00009  (0.0001) | -0.0003***  (0.00009) | -0.00008  (0.0002) | 0.00002  (0.0003) | 0.0005**  (0.0002) | -0.0001  (0.0002) | 0.0004***  (0.0001) | 0.00009  (0.0002) | -0.0004**  (0.0001) | 0.0001  (0.00010) | -0.00003  (0.0003) |
| Residual standard error | 0.124 | 0.077 | 0.056 | 0.126 | 0.177 | 0.150 | 0.146 | 0.096 | 0.155 | 0.111 | 0.062 | 0.194 |

Source: Own elaboration based on Kantar Worldpanel data.

Notes: Bootstrapped standard errors (500 replications) are in parentheses, Sig: Significance level. ***, **, * indicate significance at p <0.001, p < 0.05 and p <0.01, respectively.
