# Supplementary material for: Allocation of the household food budget among shopping basket items: How is it influenced by promotions?
Source: PLoS One. 2024 Jun 5;19(6):e0304911. doi: 10.1371/journal.pone.0304911 (PMC11152314; doi:10.1371/journal.pone.0304911)
Supplement: Attachment — Submitted filename: Response to Reviewers.docx [file pone.0304911.s002.docx]

Dear Editor,

Thank you for allowing a resubmission of our manuscript, with an opportunity to address the reviewers’ comments. We would like to thank the reviewers for the great interest they have shown in our work, as well as their relevant remarks to improve the article.

Our point-by-point response to the comments are presented below.

**Major concerns**

**Reviewer#1**

**Concern # 1:** T**he paper has not been written in an organized way; therefore, the authors need to structure the paper under these sections (or follow the PLOS ONE guidelines):** **abstract, introduction, methods (study population, theoretical framework, and statistical method), ethics, results, discussion, conclusion, and references.**

**Author response:** Thank you for your valuable feedback. We appreciate your suggestions regarding the organization of the paper. We revised the manuscript to adhere to the recommended structure, including the sections you have outlined. This restructuring enhanced the clarity and coherence of the paper, ensuring that readers can more effectively navigate and comprehend our research findings.

**Concern # 2: The introduction section needs to end up with identifying the research gap, study objective(s), followed by the three research questions.**

**Author response:** Thank you for your constructive feedback. We acknowledge the importance of clearly defining the research gap, study objectives, and research questions in the introduction section of our paper. In our revision, we ensured that the introduction concludes with a concise identification of the research gap, clearly stated study objective, and the three research questions. This provides a comprehensive understanding of the purpose and focus of our study right from the outset, facilitating better engagement with our research findings. We appreciate your guidance in refining the structure of our paper to enhance its clarity and effectiveness.

**Author action:**

The main shortcoming of the cited studies is that they restricted their analysis to a specific category of food products without considering the potential effects on the full shopping basket; that is, they assumed weak separability of the selected categories from the total food basket. These interdependencies are important because they help retailers to allocate their promotional budget across categories. Therefore, studying the full basket is a crucial component of the creation of any promotional mix in which retailers build new product bundles, provide special discounts, or put products in the best possible locations. To the best of our knowledge, very few studies have considered the effects of prices and promotions on the entire shopping basket. Drèze et al. (42) used panel data on household purchases from four stores of the same retailer, and Revoredo-Giha et al. (43) analysed the overall effect of promotions on consumers’ food purchases in Scotland by area of deprivation to formulate food and health policies. In this study, we propose a conceptual framework (Fig 2) of the effect of sales promotions and prices on household expenditures to achieve our research objective: to determine the effect of sales promotions on households’ allocation of their budget among the different products in a typical shopping basket in Spain. Our framework has two dimensions: (A) the direct effects of price and promotions on household expenditures, and (B) the possible cross-effects of price and promotions (i.e., substitution or complementarity), which could lead households to reallocate their budget.

Fig 2. Conceptual framework of this study.

Ultimately, we aim to contribute to the literature empirically and methodologically. Empirically, we aim to study household budget allocation decisions in Spain because retailers should be aware of how consumer responses to sales promotions vary between nations, states and provinces, as sales promotions are generally acknowledged as challenging to standardise due to the varying legal, economic and cultural factors that influence households and consumer behaviour. Consequently, we seek to provide retailers insights into the categories that are influenced most by promotions and how households manage their shopping budgets. Methodologically, we use the EASI demand system because it allows for flexibility of Engel curve shapes, unlike the AIDS demand model. Banks et al. (44) demonstrated, using nonparametric regressions, that Engel curves can have different shapes. Additionally, budget share error terms can be interpreted as unobserved preference heterogeneity, as permitted by the implicit Marshallian demand system developed by Lewbel et al. (45) that merges the proprieties of the Hicksian and Marshallian demand functions. In addition, we introduce promotional indices into the EASI demand system that allow for the estimation of the own-promotion and cross-promotion elasticities for a broader number of food categories.

Therefore, we will strive to answer the following three major research questions:

- To what extent do promotion and price induce households to change their shopping expenditures?
- Which product category has the strongest effect on expenditures when sold under a promotion?
- What are the possible cross-effects of promotions on the product categories in the shopping basket?

**Concern # 3:** **The study population can be presented by a flowchart, showing number of households and/or household purchases, with exclusion criteria. Currently, it is not clear whether the authors included alcoholic beverages as part of household food purchases (I don’t think alcoholic beverages can be included as part of household food basket or purchases). What other food/beverage products need to be excluded (e.g., products purchased during Eister or Christmas), or weekly household purchases less than a certain amount of money or some other products that the authors excluded or may exclude them (e.g., foods for pets).**

**Author response:** We would like to appreciate your comments, which contribute to the improvement of our article. For this question, we took into consideration the comment that you’ve raised, we added a flowchart showing the number of the households and the exclusion criteria to reach the final sample size.

We excluded household’s supplies (kitchenware, detergent, soap, insect repellent, and health and beauty care) since these categories represent a really small budget share and usually not purchased in a weekly basis which will provoke a big percentage of zeros in the dependent variable (expenditure share) of the EASI demand system.

In relation to the alcoholic beverages, they are included in the category “Drinks” as mentioned in the (S1 Appendix). As it’s a common classification in various papers; Drèze et al.(2004), and Revoredo-Giha et al.(2018) that included “Alcohol” as a category in their shopping basket analysis. And as indicated by Du and Kamakura (2008) that an in-depth analysis of the household budget allocation should include as complete a set of consumption categories as possible.

**Author Action:** Flowchart of the household selection process.

**Fig 2.** Flowchart of the household selection process

**Concern # 4:** **A theoretical framework (or conceptual framework) can be useful to guide the conceptual relationships between promotions and price and the outcome of interest, in line with the study objective(s) and/or research questions.**

**Author response:** We would like to appreciate your comments, we took into consideration the comment that you’ve raised, we added the conceptual framework of the study to show the objective and research questions of the study into the introduction.

**Author Action:**

**Fig 3.** Conceptual framework of the study

**Concern # 5:** **Also, the authors need to provide a definition for retail promotions (I guess promotion was self-reported by households in this study).**

**Author response:** Thank you for your comments, the promotions are presented in the scanner panel database are self-reported; and presented as a categorical variable, that takes the value of 1 if the household’s made a transaction with a promotion and takes a value of 0, otherwise. This is a common case for all the scanner panel data as mentioned in Muth et al., (2019), that households in scanner panel data indicate whether they used some kind of promotion.

**Author Action:**

To identify and track products, a Universal Product Code (UPC) is employed, where each household were equipped with a barcode reader that allowed them to scan the UPCs and report all the information present in their shopping ticket; quantities, prices and a promotional variable that refers to retailers’ promotions, presented as a categorical variable that takes the value of 1 when the household purchase under promotion and 0 otherwise. The dataset also includes product characteristics (such as type of packaging, brand and flavour), and some socio-demographic characteristics of the household such as age, social class, presence of children, region, etc.

**Concern # 6:** **Statistical methods: The outcome of interest, predictors, and statistical models (the expenditure regression, and EASI) need to be fully described. Some formulas, which are not in line with the research questions, can be moved to supplementary materials (e.g., the formula on weighted average price). In the regression model, variables on socioeconomic status and demographic of households need to be included, in addition to the variables on promotion and price, because currently the expenditure regression does not account for such important explanatory variables (for example, household food expenditures can be affected by household size).**

**Author Response:** Thank you for the valuable comment, the formulas in relation to the data preparation (category price index, promotion index with category expenditures have been moved to the supplementary materials).

In relation to the regression model; the socioeconomic status and demographic of the households couldn’t be included in the regression as mentioned in the paper, that it is a fixed effect regression model. We added more explanation below the model to explain the fixed effect regression and the Hausman test that sustained why we choose the fixed effect regression over random effects.

### Author Action:

### Effects of promotion on the shopping food expenditures.

For assessing the impact of promotion and price on the total amount spent by households in each shopping trip *(X_ht_)*, the following panel data regression model is specified:

${lnX}_{ht}=\alpha_{0}+\sum_{g=1}^{13} b_{g}{lnp}_{hgt}+\sum_{g=1}^{13} c_{g}{Pm}_{hgt}+r_{t}^{(h)},$	(1)

Where $r_{t}^{\left( h \right)}=\rho^{\left( h \right)}+u_{t} , u_{t \sim} i.i.d. N \left( 0, \sigma_{u}^{2} \right)$

$ln$ denotes the natural logarithm, since the model is presented under the log-log form, after which the price coefficient will be interpreted as ‘elasticity’; *α_0_*, *b_g_* and *c_g_* are the regression coefficients related to each variable; *g* is the food group number (1, 2, ..., 13); *p_hgt_* and *Pm_hgt_* are the price and promotion indices, respectively. The symbol $u_{t}$ is the idiosyncratic error that varies over time, and *ρ^(h)^* is the time constant unobserved heterogeneity that represents the fixed-effect specification. According to Hun (49), the fixed-effect model assumes the same slope and a constant variance across individuals; and since the household characteristics cannot vary over time, *ρ^(h)^* is allowed to be correlated with the other regressors. Then, the Hausman specification test was used to check which of the fixed-effect or random-effect models was the most appropriate for this analysis under the null hypothesis that individual effects are uncorrelated with any regressor in the model (49). In our case, the null hypothesis of no correlation was rejected (*X*^2^_0.000,26_ = 157.92), showing that the fixed-effect model is the most appropriate for the estimation (1).

**Concern # 7: The results should present baseline characteristics of households as well as frequency of household food purchases (by food categories, prices, quantities, etc.), followed by results in response to each of the research questions in tables/figures that are well organized and clearly presented (the authors can have a look at the published papers for examples of tables and figures). The authors should use terms that are easily understood and are meaningful to a general audience of the paper (all readers are not econometricians to understand coefficients, significance shown by ***, or other technical jargons).**

**Author Response:**

1. Thank you for the comments, they have been taken into consideration. We added average number of shopping trips, average price and quantity. We added a discussion of the added statistics too.

2. A note indicating the meaning of the stars is added in bottom of each table. Additionally, the standard errors are added in a new column.

**Author Action:**

Table 1 shows some descriptive statistics related to the 13 food categories. These statistics include the average number of shopping trips, the average shopping quantity, average price, mean budget share, expenditure per shopping trip, percentage of shopping trips with promotions, category expenditure when at least one product was on promotion and expenditures without any promotion.

As can be observed, on average, households tend to purchase ‘Fruit, fruit products and fruit and vegetable juices’, ‘Drinks’ and ‘Grains and grain-based products’ by a frequency of 16.35, 16.08 and 15.10 times, respectively. The least purchased categories are ‘Snacks and other foods’ and ‘Starchy roots, tubers, legumes, nuts and oilseeds’.

Regarding quantities, ‘Drinks’ is the category that is purchased in the highest quantities (6.63 l), followed by ‘Fruit, fruit products and fruit and vegetable juices’ (2.15 kg). The smallest quantities are observed for ‘Snacks and other food’ and ‘Starchy roots, tubers Legumes, nuts and oilseeds’. We also see that ‘Fish and seafood’, with ‘Meat’, have the highest price (€2.26 and €1.54, respectively), while the lowest prices are observed for ‘Fruit, fruit products and fruit and vegetable juices’ and ‘Milk, dairy products and milk product imitates’. The highest share of the household’s budget is allocated to ‘Drinks’ (14.21%), followed by ‘Meat’ (11.81%), ‘Fish and other seafood’ (10.29%) and ‘Sugar and confectionery and prepared desserts’ (10.29%). A smaller budget is allocated to ‘Starchy roots, tubers legumes, nuts and oilseeds’ (1.97%) and ‘Snacks and other food’ (2.48%).

Concerning the percentage of shopping trips made by the households that included a promotion, it ranged from 21.47% for ‘Grains and grain-based products’ to 50.56% for ‘Fruit, fruit products and fruit and vegetable juices’. For this same metric, we note that the percentage exceeds 40% for most food categories (drinks, snacks and other food, sugar and confectionery and prepared dessert, composite dishes, and fish and other seafoods). These high percentages of promotions lead to an increase in categories’ expenditure, as shown in Table 1. Expenditures increase when promotions are used for all the categories. On average, households spend 55.54€ per shopping trip in the absence of promotion, and it increases by 5.91% when they buy at least once under promotion to get to 58.82€. However, such a rise is heterogeneous among food categories. For instance, ‘Vegetables and vegetable products’, ‘Fish and other seafood’ and ‘Drinks’ show the highest change in expenditure (114.24%, 108.61% and 100.87%, respectively), while the expenditure for ‘Sugar and confectionery and prepared desserts’, ‘Grains and grain-based products’ and ‘Snacks’ vary from 91.54% to 97.25%.

**Table 1.** **Food group statistics.**

| **Food category** | **Shopping frequency** | **Average quantity** (kg/l) | **Average price**  (€) | **Mean budget share** (%) | **Percentage of trips with promotion** (%) | **Average expenditure without promotion in the shopping trip** (€) | **Average expenditure with promotion in the shopping trip** (€) | **Percentage change between expenditures** (with & without promotion; %) |
| --- | --- | --- | --- | --- | --- | --- | --- | --- |
| Grains & grain-based products | 15.10  (10.58) | 0.54  (0.81) | 0.89  (1.10) | 5.56 | 21.47 | 2.49  (2.27) | 4.85  (3.82) | 94.78 |
| Vegetables & vegetable products | 13.41  (11.23) | 0.90  (2.25) | 0.73  (1.31) | 3.89 | 29.56 | 3.02  (2.73) | 6.47  (5.32) | 114.24 |
| Starchy roots, tubers, legumes, nuts & oilseeds | 11.08  (9.47) | 0.24  (0.66) | 1.25  (2.63) | 1.97 | 24.73 | 2.85  (2.42) | 4.62  (4.28) | 62.11 |
| Fruits, fruit products, & fruit & vegetable juices | 16.35  (11.88) | 2.15  (3.61) | 0.42  (0.62) | 8.05 | 50.56 | 4.36  (4.47) | 7.63  (6.38) | 75.00 |
| Meat | 14.62  (10.60) | 0.52  (1.03) | 1.54  (2.10) | 11.81 | 32.63 | 10.83 (13.07) | 12.68 (16.19) | 17.08 |
| Fish & other seafoods | 12.61  (9.06) | 0.57  (1.11) | 2.26  (3.85) | 10.29 | 41.84 | 8.36 (7.94) | 17.44 (15.22) | 108.61 |
| Milk, milk products & milk imitation products | 14.99  (9.78) | 2.89  (5.29) | 0.54  (0.67) | 8.79 | 31.49 | 5.16 (5.27) | 8.51 (7.46) | 64.92 |
| Cheese | 13.25  (9.92) | 0.28  (0.53) | 1.67  (1.97) | 5.59 | 28.27 | 4.60  (4.37) | 8.38  (8.20) | 82.17 |
| Sugar, confectionery & prepared desserts | 14.51  (10.87) | 0.70  (1.09) | 1.40  (1.60) | 10.29 | 45.73 | 4.49  (3.98) | 8.60  (7.59) | 91.54 |
| Composite dishes (animal & vegetable) | 13.57  (10.71) | 0.64  (1.36) | 1.65  (2.92) | 7.24 | 43.41 | 5.57  (4.85) | 10.42  (7.85) | 87.07 |
| Snacks & other foods | 9.78  (8.66) | 0.12  (0.26) | 1.61  (2.18) | 2.48 | 46.85 | 2.18  (1.71) | 4.30  (2.63) | 97.25 |
| Drinks | 16.08  (11.04) | 6.63  (11.38) | 0.56  (1.23) | 14.21 | 49.95 | 6.87  (7.16) | 13.80 (13.48) | 100.87 |
| Residual category | 13.57  (10.71) | 0.77  (0.77) | 2.66  (4.52) | 9.82 | 36.69 | 7.00  (8.11) | 11.86 (12.50) | 69.43 |
| Expenditure per shopping trip |  |  |  |  |  | 55.54 (40.16) | 58.82 (40.81) | 5.91 |

2. **Table 3. Estimation results of total expenditure regression.**

| Variables | Coef. | SE | Sig |
| --- | --- | --- | --- |
| Intercept | 3.286 | 0.037 | *** |
| ***Prices*** |  |  |  |
| Grains and grain-based products | 0.051 | 0.013 | *** |
| Vegetables and vegetable products | 0.029 | 0.013 | ** |
| Starchy roots, tubers, legumes, nuts and oilseeds | 0.039 | 0.015 | *** |
| Fruit, fruit products and fruit and vegetable juices | 0.092 | 0.015 | *** |
| Meat | 0.115 | 0.015 | *** |
| Fish and other seafood | 0.107 | 0.017 | *** |
| Milk, dairy products and milk product imitates | 0.043 | 0.015 | *** |
| Cheese | 0.104 | 0.021 | *** |
| Sugar and confectionery and prepared desserts | 0.072 | 0.011 | *** |
| Composite dishes (animal and vegetable composite dishes) | 0.041 | 0.011 | *** |
| Snacks and other food | 0.125 | 0.12 | *** |
| Drinks | 0.082 | 0.14 | *** |
| Residual category | 0.004 | 0.12 |  |
| ***Promotions*** |  |  |  |
| Grains and grain-based products | 0.359 | 0.124 | *** |
| Vegetables and vegetable products | 0.430 | 0.144 | *** |
| Starchy roots, tubers, legumes, nuts and oilseeds | 0.407 | 0.117 | *** |
| Fruit, fruit products and fruit and vegetable juices | 0.802 | 0.141 | *** |
| Meat | 0.694 | 0.140 | *** |
| Fish and other seafood | 0.858 | 0.112 | *** |
| Milk, dairy products and milk product imitates | 0.602 | 0.107 | *** |
| Cheese | 0.400 | 0.129 | *** |
| Sugar and confectionery and prepared desserts | 0.705 | 0.126 | *** |
| Composite dishes (animal and vegetable composite dishes) | 0.806 | 0.113 | *** |
| Snacks and other food | 0.125 | 0.098 |  |
| Drinks | 0.655 | 0.081 | *** |
| Residual category | 1.046 | 0.099 | *** |
| N | 3564 |  |  |
| R^2^ | 0.326 |  |  |

Source: Own elaboration based on Kantar WorldPanel data.

Notes: Coeff: Coefficient, SE: standard error, Sig: Significance level. ***, **, * indicate significance at p <0.001, p < 0.05 and p <0.01, respectively.

**Concern # 8:** **Discussion: The first paragraph may cover an overall summary of the findings in the study, followed by comparison of the findings with relevant literature and possible explanations for discrepancies and similarities in the findings, with references. The study limitations and strengths, and the findings’ significance on public health nutrition policies can be discussed. Further research highlighted.**

**Author Response:** Thank you for your thoughtful feedback. We appreciate the opportunity to address the comments regarding the discussion section of our study. In response to the reviewer's suggestion, we have revised the discussion section to provide a more comprehensive overview of our findings and their implications. Firstly, we have included an overall summary of the key findings of our study. Secondly, we have incorporated a comparison of our findings with existing literature. Additionally, we have discussed possible explanations for any disparities observed between our findings and those of previous studies. Furthermore, we have critically examined the limitations of our study. By acknowledging the constraints and biases inherent in our research design, we aim to provide a transparent assessment of the reliability and validity of our findings.

Importantly, in relation to the discussion of the significance of our findings for public health nutrition policies. We tried to drive some conclusions, but the classification of the food categories from the beginning was not intended for studying the effect of promotion on the healthy eating and purchasing behavior. But we consider it as an interesting idea for future research.

Overall, we believe that this revision enhances the discussion section of our study, providing a more thorough analysis of our findings and their implications. We are grateful for the reviewer's insightful feedback, which has helped to strengthen our manuscript.

**Author Response:**

Discussion

In this study, we present estimates of the impacts of both price and promotion on household expenditures for all items in the shopping basket, as well as on the allocation of the shopping budget across the shopping basket items. To do so, we used two complementary quantitative tools. First, we estimated a fixed-effect regression model to study the impact of price and promotion on household expenditures in our selected retailer outlets. Second, we used the censored EASI demand system established by Lewbel and Pendakur (45) to calculate the expenditure, price and promotion elasticities in order to analyse the cross-product effects of price and promotion, considering the censored nature of the dependent variable, which, in this study, was the expenditure share.

The results of the fixed-effect regression indicate that both price and promotion had a positive effect on the household expenditures. The price elasticities were lower than unity, indicating the price-inelastic nature of the food products and that the households were not price-sensitive. This result further indicates that the food products were weakly separable from each other. This result is in line with that of Revoredo-Giha et al. (43) and in contrast with the result of Drèze et al. (42), which showed a negative effect of price on household expenditures and more price-sensitive consumers. These contrasting results are probably due to the different countries studied and the difference in the product aggregation, which, in (42) , was focused on the retailers’ product assortment, and in the current study, was based on the Spanish nutrition guidelines.

As for promotion, various studies (41,52) reported its positive impact on household expenditures, as explained by the retailers’ promotional objective of enhancing their establishments’ sales in other words, pushing households to spend more while shopping.

The results of the EASI demand system showed that demand for all the food categories, except for the ‘Residual category’, was inelastic in response to price changes, similar to (53,54) in Vietnam, (55) in the USA and (56) in Spain. ‘Drinks’ showed the highest own-price elasticity, as mentioned in (43) and (57). Contrary to the findings of (56,58) in Spain, ‘Milk, milk products and milk imitation products’ had the second highest elasticity, similar to (42,57,59,60). In any case, these comparisons are not straightforward, as the food aggregations differ across studies.

As for the cross-price elasticities in this study, many of them were quite low and insignificant. Moreover, the most significant cross-price elasticities revealed complementary relationships, as we expected, considering the broad number of food categories we considered. Substitution relationships were expected in each food category, while complementarity or even independent relationships were expected between categories, in line with (55). In any case, the cross-price elasticities were much smaller in absolute value relative to the own-price elasticities, as in Molina (61).

From the expenditure elasticities, we saw that all the food categories behaved as normal goods, which is also quite consistent with our expectations, as we are dealing with food categories and not with specific food products, among which we could expect some inferior goods behaviour. After the ‘Residual category’, the ‘Grains and grain-based products’, ‘Meat’, ‘Milk, milk products and milk imitation products’ and ‘Fish and other seafoods’ categories had the highest sensitivity to expenditure changes, as they were elastic. These results are supported by other studies in Spain (57,58) for ‘Milk, milk products and milk imitation products’ and by (56,60,62) for ‘Meat’ and ‘Fish and other seafoods’. Other studies outside Spain reported similar findings (54,63,64).

Promotion had a positive effect on the household expenditures in this study, even though the values were relatively small. The values for ‘Composite dishes’, ‘Sugar, confectionery and prepared desserts’, ‘Fish and other seafoods’ and ‘Drinks’ were the highest. These results are quite similar to those of (43), that in which promotion had the strongest effect on unhealthy products, such as beverages, soft drinks and confectionery, while the elasticity of the ‘Meat’ category was among the smallest across the categories. In any case, the values we obtained were higher. Differences in the econometric model, the country of study or the aggregation level of food products could have caused such discrepancies.

The negative spillover effects of promotions is the predominant pattern in this study, as many of the cross-promotion coefficients were negative, which indicates that an increase in promotion in one category decreased the demand for the other categories of the shopping basket. This behaviour is quite intuitive; as promotion induces a higher demand for the promoted product, households will switch to the non-promoted category with the promoted one, which will provoke a small but significant budget allocation between categories in the shopping basket. However, we also found a statistically significant indication of a positive spillover effect of the promotion of ‘Fruit, fruit products, and fruit and vegetables juices’ on the demand for ‘Vegetables and vegetables products’. These cross-effects are asymmetric between related categories, as Manchada et al. (35) also found, although in a non-food context where they studied the effects of price changes and promotions on grocery categories (i.e., laundry detergents, fabric softeners, cake mixes and cake frostings). Similar findings have been reported by William et al. (65) with Martinez-Ruiz et al. (66) for soft drinks and coffee respectively.

These studies reported that the own-promotion effects were stronger than cross-effects and that there was an asymmetric promotion cross-effect between pairs of categories.

Our results can contribute to improving retailers’ understanding of consumers’ reactions to promotion strategies for different food categories in Spain. While retailers analyse the impact of their promotions’ strategies on a specific promoted food product, cross-effects on non-promoted categories are not considered, which can be negative. Although such negative effects are of lower magnitude than the own-effect, the accumulated negative effect on the rest of the categories should not be underestimated. That is, retailer managers in Catalonia should promote categories such as ‘Fish and other seafoods’, ‘Composite dishes’, ‘Fruits, fruit products, and fruit and vegetable juices’, ‘Sugar, confectionery and prepared desserts’ and ‘Drinks’ with caution as to the cross-effects.

From a health perspective, although this was not the focus of this study, our results indicate that promotions for healthier food categories, such as ‘Fruit, fruit products, and fruit and vegetable juices’ and ‘Vegetables and vegetable products’ had a negative effect on ‘Snacks and other foods’, ‘Sugar, confectionery and prepared desserts’ and ‘Drinks’. However, the reverse effect was also seen; that is, promotions for ‘Drinks’ decreased the budget share of most of the categories, including of ‘Fruit, fruit products, and fruit and vegetable juices’ and ‘Vegetables and vegetable products’.

In any case, our results should be interpreted with caution, as they refer to a specific geographical area and to a specific retailer chain with heterogeneous food categories.

In our research paper, it is crucial to acknowledge certain limitations that may affect the scope and applicability of our findings. One significant limitation of our paper is the reliance on data collected from 2017, that may not accurately reflect the current state of markets, trends, and behaviors that could have been changed especially after COVID 19. Additionally, extending this study to other areas and retailers with different business profiles could be challenging. Furthermore, our dataset reported promotions as a categorical variable, therefore we couldn’t estimate their intensities.

While our study has provided valuable contributions to the current understanding of the topic at hand, there remain several promising directions for future research. From a food policy perspective, it could be interesting to differentiate between promotions for healthy and unhealthy products and to check if promotions for food products in each category contribute to a healthier or unhealthier diet, considering the total food expenditure. Either way, this potential future research should start with a proper definition of what is meant by ‘healthy’ and ‘unhealthy’ and with a reclassification of food categories according to this distinction. And by using recent data, we can delve into the consumer reactions towards sales promotions during COVID-19 and afterwards.

**Concern#9: Conclusion: The overall interpretation of the findings in the context of Catalonia (or Spain) or wider region and world can be described. Recommendations may be suggested considering the findings.**

**Author Response:** Thank you for your valuable feedback on the conclusion section of our study. We have carefully considered your suggestions and have made the necessary revisions. However, as mentioned in the guidelines of the PLOS ONE journal the section of discussion can be joined to the conclusion “*These sections may all be separate, or may be combined to create a mixed Results/Discussion section (commonly labeled “Results and Discussion”) or a mixed Discussion/Conclusions section (commonly labeled “Discussion”). These sections may be further divided into subsections, each with a concise subheading, as appropriate. These sections have no word limit, but the language should be clear and concise. Together, these sections should describe the results of the experiments, the interpretation of these results, and the conclusions that can be drawn*”. So, we made the necessary changes in the discussion section.

**Minor issues**

**Concern#10: English language needs substantial improvement throughout the paper.**

**Author Response:** Thank you for the comments regarding the English, the manuscript has been revised and proofread by an external company and the certificate of proofreading is joined during the resubmission process. Nonetheless, we made sure to revise it again for any mistake that could have been missed.

**Concern#11: There are technical jargons that the authors may need to provide a brief definition of them whenever they are used the first time in the paper (e.g., micro-panel in line 16).**

**Author Response:** Thank you for the comments, the manuscript has been checked and we made sure that all the technical words have been defined at least once when they are mentioned for the first time.

**Author Action:** The micro-panel has been replaced by “scanner panel data” which will be more convenient to understand.

The empirical analysis was performed using consumer scanner panel data spinning from January 2017 to December 2017. The data were obtained from the ©Kantar Worldpanel Company’s network in Spain and provide detailed shopping information from a representative random sample for Spain.

**Concern#12: The authors used some unnecessary words in some parts of the paper which can be confusing. For example, from lines 132-133 “Thus, three major research questions which are worth to be answered represent the objectives of our study.”. The authors can use a clear and concise sentence, instead. For example, they can use “In line with the study objectives, three research questions were examined.”**

**Author Response:** Thank you for the recommendation, we made sure to revise any confusing expression and changed through the manuscript.

**Concern#13: What is shopping ticket expenditure? (For example, in line 279 and elsewhere). Please use key terms consistently throughout the paper, as currently it is not clear whether “shopping ticket expenditure” means “shopping food expenditure” or something else.**

**Author Response:** Thank you for your comments, the two expressions refer to the households’ expenditures but we made sure to use one expression to refer to the households’ expenditures which is “Shopping food expenditure”

**Concern#14: The authors stated that zero purchases are relatively frequent in their data sets (lines 317-318). Is there a way that the authors can use to avoid “zero purchases”, because currently it seems (from the way the authors coded household purchases) that it was assumed that a household would purchase all food categories in each shopping trip (lines 317-318) which does not seem logical.**

**Author Response:** Thank you for your comments, maybe we were not sufficiently clear in our approach explaining how we dealt with zero purchases problem. We didn’t assume that a household would purchase all the food categories in the shopping trip, but we divided the customer journey into two major steps as in (Heien and Wessells, 1990). Where in the first step the household decide to consume a specific category or no as a function of his preferences, shopping expenditures, and prices using a probit model. From the results of this first model, an Inverse Mills Ratio (IMR) which is equal to [ratio](https://en.wikipedia.org/wiki/Ratio) of the [probability density function](https://en.wikipedia.org/wiki/Probability_density_function) to the [cumulative distribution function](https://en.wikipedia.org/wiki/Complementary_cumulative_distribution_function) of a distribution is introduced in the EASI demand system to solve the problem of censoring. In any case, we will add the model and all the related formulas to the appendix together to the formulas used in the data preparation.

**Concern#15: There is a contradiction in the findings in lines 405-407 “…except for the ‘Residual category’, which includes a wide range of quite heterogeneous products, all food categories are price inelastic.” with those in line 408 “As can be observed, the category of ‘Drinks’ shows the highest own-price elasticity…” The authors need to check such issues throughout the paper.**

**Author Response:** We thank you for the comment, it’s true maybe it may create a confusion while reading the paper, but what we meant by “As can be observed, the category of ‘Drinks’ shows the highest own-price elasticity…” is that between the inelastic categories, Drinks shows the highest value. But we took into consideration comment and we made the necessary changes throughout the paper.

**Reviewer#2,**

**Concern#1: The overarching objective of the study is to evaluate the impact of retail promotions on the overall expenditure in shopping baskets. However, the introduction section currently narrows its focus to a specific aspect, overlooking other potential effects that advertisements may exert on consumers. This omission hinders a comprehensive review of relevant studies and their implications. It's important to note that the effectiveness of retail promotions can vary based on factors such as the type of promotion, the target audience, market conditions, and the overall economic environment**.

**Author Response:** Thank you for your insightful comment regarding the focus of the introduction section of our study. We appreciate your perspective and acknowledge the importance of providing a comprehensive overview of the potential effects of retail promotions on consumer behavior. In response to your feedback, we broadened the scope of the introduction section to encompass a more holistic examination of the impact of retail promotions on consumer spending behavior. By considering a wider range of factors such as the type of promotion, and the possible promotion effects that could be generated by promotions.

**Author Action:**

Considering the short term, various studies have focused on the influence of various promotions on consumer perception and purchasing behaviour, and on company profitability and performance. Boto et al. (17) and Van Lin et al. (18) maintained that promotions induce an increase in sales of the promoted products. Ramanathan and Dhar (19) pointed out that aside from promotions’ positive own-effect, they can push consumers to buy non-promoted products. Other studies claimed that sales promotions can induce brand switching (8,20–22) or generate a cannibalisation effect (23). Some other studies have focused on which promotional tools are most effective. For instance, Obeid (24) found that price discounts and more free products could have the greatest impact on consumer purchase behaviour. Moreover, Vigna and Mainardes (25) demonstrated that discounts induce purchase acceleration, stockpiling and product trials.

**Concern#2: The research methodology effectively elucidates various intricacies; nevertheless, a crucial point of contention lies in the justification of utilizing dated data from 2017. The authors must substantiate how the household food budget, consumer behaviour, and advertising methods have remained unchanged since then. This is essential to ensure the article's relevance and appeal to contemporary readers. It also needs to be mentioned as a limitation of the research at the end of the conclusion.**

**Author Response:** Thank you for your insightful feedback regarding the utilization of data from 2017 in our research methodology. We understand your concern about the relevance of the data and its implications for the study's findings and broader applicability to contemporary readers. We acknowledge that using data from 2017 may raise questions about its current relevance, particularly in an ever-evolving landscape of consumer behavior and advertising methods. Therefore, we provide a robust justification for our decision to use it. Our rationale for selecting the 2017 data is based on several factors; firstly, the availability of this data in the moment of the analysis knowing that this kind of data is expensive. Second, as we can observe from the (Fig 1), the expenditure share of food in the households remained more or less unchanged from 2017 to 2019 and ranges from 14.1 to 14.2 % of the household budget. After 2019, changes occurred in the consumer behavior mainly because of the pandemic. The same is observed in relation to the promotional investment in Spain (Fig 2), where we don’t observe a big difference between the years starting from 2016 until 2019, a year before COVID-19. From 2020, things have changed.

Furthermore, we addressed the limitations associated with using dated data in the discussion and conclusion section of the manuscript. By acknowledging this limitation, we aim to provide transparency about the potential impact on the generalizability and applicability of our findings to current contexts.

|  | Euros | Percentage (%) |
| --- | --- | --- |
| 2013 | 74639.9 | 15.1 |
| 2014 | 73729 | 14.9 |
| 2015 | 75802.1 | 15.1 |
| 2016 | 76050 | 14.6 |
| 2017 | 76042 | 14.1 |
| 2018 | 78670.8 | 14.1 |
| 2019 | 80328.8 | 14.2 |
| 2020 | 86289.3 | 17 |
| 2021 | 90668.4 | 16.4 |
| 2022 | 96127.8 | 16 |

**Fig 1. Total annual expenditure of households 2013-2022 (€)** (“Instituto de Estadística de Cataluña,” 2024)

|  | Total |
| --- | --- |
| 2013 | 2159.7 |
| 2014 | 2220.1 |
| 2015 | 2290 |
| 2016 | 2275.1 |
| 2017 | 2256.5 |
| 2018 | 2293.5 |
| 2019 | 2246.7 |
| 2020 | 1670.6 |
| 2021 | 1797.3 |
| 2022 | 1848.7 |

**Fig 2. Investment on advertising in Spain 2013-2022 (M€)** (Eguinoa, 2022)

**Concern#3: This concern extends to the references, as a substantial portion of them pertains to older works. To enhance the article's current appeal and scholarly robustness, it is essential to incorporate recent research findings and participation, thereby fortifying the foundation of the study with up-to-date and pertinent insights.**

**Author Response:** Thank you for your thoughtful feedback on our article. We appreciate your insight regarding the need to enhance the current appeal and scholarly robustness of our work by incorporating recent research findings. We understand the importance of updating references with recent research to fortify the foundation of our study with up-to-date and pertinent insights. We carefully reviewed our references and ensure that we integrate recent research findings that contribute to the advancement of our field.

**Reviewer#3**

**Concern#1: This is a good paper. I think the analysis is sound and general approach is sound and appropriate. The authors addressed the objectives of the paper with commonly used methods, addressing concerns of endogeneity and other issues to my satisfaction. The paper is well written and has clearly stated objectives. Well, done.** **I do have some minor comments and questions: I do not think it is clear that food retail competition has "increased" as the authors claim in several places in the paper. If anything, food retail has consolidated, reducing concentration in some countries. I would like the authors to support this claim with an example or statistic like a CR4 ratio or HHI data. I suspect that competition has declined and that in an environment with fewer firms, differentiation is the optimal strategy.**

**Author Response:** Thank you for the comment, the market of retailers in Spain is quite competitive, According to Statista (Statista, 2024). Since the CR4 refers the market share of the four largest firms in the market, we calculated the CR4 ratio and it’s equal to 47.5% which indicates a monopolistic Competition (Naldi and Flamini, 2014). In this case it refers to a market structure characterized by a differentiated product and freedom of entry and exit. Differentiated products provide each firm with some market power. Advertising and marketing of each individual product provide uniqueness that causes the demand curve of each good to be downward sloping. Product differentiation is the key feature of monopolistic competition, where products are marketed by quality or brand. Demand is highly elastic, and any change in pricing can cause demand to shift from one competitor to another.

However, we changed the first sentence of our introduction to “Due to the monopolistic competition in the retail market in Spain”.

**Concern#2: I am assuming, these "promotions" are not price reductions but rather advertisement and similar marketing activities. Can you measure the "intensity" of these marketing events somehow? currently it looks like you are using a yes/no variable. I would like to see the intensity reflected, if possible.**

**Author Response:** We would to thank you for the relevancy of the comment, unfortunately the promotion is represented as a dummy variable in the database, that takes a value of 1 if there is a promotion and 0 otherwise, independently of the type of promotion. Therefore, we can’t reflect the intensity of promotion in our research. But we made sure to mention it as a limitation in our work

**Concern#3: Line 234 and other places have an error message. Also, check equation numbers, they may be off (did you skip 7?) Double check your math notation and order of terms, especially eq. 6 and 8.**

**Author Response:** Thank you for the comments, in relation to the error message this may be due the fact that the submitted file was a word file and some errors appeared.

In relation to the equation numbers, yes, the number 7 have been skipped and we made sure to check the numbers again. In relation to the order of terms and math notations they have been revised too.

**Author Action:**

To analyse the effects of promotions on the demand for the 13 food categories, we specified the EASI demand model (45). As already stated, the model allows for more flexible Engel curves and for interpreting the error terms as unobserved household preferences. The model is as follows:

$w_{hgt}=\sum_{r=1}^{L} B_{gr}y_{ht}^{r}+\sum_{g=1}^{G} A_{ig}lnp_{hgt}+{CPm}_{hgt}+{Dz}_{n}+\varepsilon_{hgt}$	(2)

for $h=1,\cdots,H;g=1, \cdots, G;and t=1, \cdots, T$,

where *w_hgt_* is the expenditure share of the food category *g* for household *h* in period *t*; *G* is the number of food categories; *H* is the number of households; *lnp_hgt_* is the vector of the logarithmic price indices; *y_ht_* is the log total real expenditure (the curvature of the Engel curve) which is calculated by deflating the nominal total expenditure $X_{ht}$ with the Stone price index, as follows:

$y_{ht}={lnX}_{ht}-\sum_{g=1}^{G} {lnp}_{hgt}w_{hgt}$, (3)

*L* is the highest degree of polynomials of *y_ht_* to be determined numerically; *Pm* is a vector of the promotional indices; *z* is an *n* vector of the sociodemographic characteristics; *ԑ* is the error term that captures the unobserved heterogeneity; and *A*, *B*, *C* and *D* are the coefficients to be estimated.

To determine the optimal degree of the polynomial ($L)$ in (2), the model was estimated sequentially starting with *r* = 1 up to *r* = 5, and a joint test of the significance of the parameter *B_gr_* was performed. The *p*-value was <0.00 for *r* = 3, indicating that the cubic functional form was the most appropriate in this case to capture the curvature of the Engel curves.

As not all the households purchased food from all the categories in each shopping trip, zero purchases were relatively frequent in our data set. To account for the censored nature of the dependent variable, we followed the two-step procedure suggested by Heien and Wessells (50). In the first step of this procedure, the decision to purchase is modelled as a dichotomous choice problem that has a value of 1 if a household consumes food in a category during a shopping trip and 0 otherwise. Then, the regression is used to compute the inverse Mills ratio (IMR) for each household, which is equal to the [ratio](https://en.wikipedia.org/wiki/Ratio) of the [probability density function](https://en.wikipedia.org/wiki/Probability_density_function) to the [cumulative distribution function](https://en.wikipedia.org/wiki/Complementary_cumulative_distribution_function) of a distribution. The detailed model is presented in the supplementary material (S1 Appendix). Then, the IMR for each food category is used as an instrument in the second step of the demand estimation, as follows:

$w_{hgt}=\sum_{g=1}^{G} A_{ig}lnp_{hgt}+\sum_{r=0}^{3} B_{r}y_{ht}^{r}+{CPm}_{hgt}+{Dz}_{n}+{\delta R}_{hgt}+\varepsilon_{hgt}$, (4)

where, all the variables are the same as the ones stated in equation (2), $R_{hgt}$ is the instrument referring to the calculated IMR.

Adding-up and homogeneity were imposed by the introduction of the following restrictions:

$1_{g}^{´}b_{0}=1, 1_{g}^{´}b_{r}=0 \forall r\neq0,$	(5)

and

$1_{g}^{´}A=1_{g}^{´}C=0_{g}, 1_{n}^{´}D=0_{n}$, (6)

Moreover, Slutsky symmetry is guaranteed by the symmetry of the $g \times g$ matrices of$A$.

1.1.1 Endogeneity

Two sources of endogeneity can arise from the demand system equation (4). The first is related to the presence of budget shares in the Stone price index. This kind of endogeneity can be negligible, according to Zhen et al. (51) and Lewbel and Pendakur (45). The second source of endogeneity is related to the real food expenditure (*y_ht_*), which is a function of the nominal total expenditure (*X_ht_*). To account for the latter, we followed Lewbel and Pendakur (45) by using *y̅_ht_* as an instrument for real food expenditure, which was estimated by replacing $w_{ht}$ with its mean, *w̅_ht_*, in the following equation:

$\bar{y}_{ht}={lnX}_{ht}-\sum_{g=1}^{G} {lnp}_{hgt}\bar{w}_{g}$ , (7)

The model was estimated using the three-stage least squares (3SLS) method to account for endogeneity. The expenditure elasticities, Hicksian and Marshallian price elasticities with promotional elasticities, were estimated following (41,44,51), as described next.

The matrix for the compensated Hicksian price elasticities for a food category *g* with respect to the price of food category *j* is given by:

$\epsilon=\bar{w}^{-1}\left( A \right)+\Omega\bar{w}-I$ , (8)

where ϵ is *n × n* of the compensated demand elasticities, *w̅* is an identity matrix where the ones have been replaced by the mean of the budget shares, *A* is the *n × n* matrix of price coefficients, Ω is an *n × n* matrix of ones, and *I* is an identity matrix.

The vector of the expenditure elasticities (*ϑ*) was calculated using the following equation:

$\vartheta=\bar{w}^{-1}{(I+\Theta\acute{p)}}^{-1}\Theta+1_{g}$ , (9)

where *ϑ* is a vector of *G × 1* of the estimated expenditure elasticities; *Θ* is a vector of expenditure semi-elasticity coefficients, which is $\sum_{r=0}^{3} rB_{r}y^{r-1}$; $p$ is a vector of the mean prices; and *1_j_* is a *G × 1* of ones.

The matrix of uncompensated Marshallian elasticities (*θ*) was derived from the Slutsky equation using the following equation:

$\theta= \epsilon-\bar{w}\vartheta$ , (10)

The matrix of promotional elasticities, *ω*, was derived as follows:

$\omega=\bar{w}^{-1}\left( C \right)*\bar{P}_{m}$ , (11)

where *w̅* is an identity matrix where the ones have been replaced with the means of the budget shares; *C* is the *n × n* matrix of promotional coefficients; and *P̅_m_* is an identity matrix where the ones have been replaced by the mean of the promotional indices.

**Concern#4: The specification in equation 5 makes sense to me. One question I have is about the potentially confounding effect of time. You have done well to include individual fixed-effects. Is there a reason not to include some sort of time based fixed-effect? Perhaps there is a seasonal component to expenditures that you are not accounting for--such a holiday season where both promotions and holiday traditions increase expenditure. You would want to disentangle these effects**.

**Author Response:** We thank you for the comments. Actually, we added the time effects in the regression model but it was not significant so we took them out form the model. Below you can see the results of the regression including the time effect.

| lnX | Coef. | Std. Err. | P>t |
| --- | --- | --- | --- |
| *lnPC1* | 0.053 | 0.014 | 0.000 |
| lnPC2 | 0.036 | 0.013 | 0.006 |
| lnPC3 | 0.063 | 0.017 | 0.000 |
| lnPC4 | 0.094 | 0.015 | 0.000 |
| lnPC5 | 0.113 | 0.015 | 0.000 |
| lnPC6 | 0.126 | 0.016 | 0.000 |
| lnPC7 | 0.048 | 0.015 | 0.002 |
| lnPC8 | 0.119 | 0.018 | 0.000 |
| lnPC9 | 0.070 | 0.015 | 0.000 |
| lnPC10 | 0.044 | 0.016 | 0.005 |
| lnPC11 | 0.145 | 0.023 | 0.000 |
| lnPC12 | 0.084 | 0.011 | 0.000 |
| lnPC13 | 0.006 | 0.011 | 0.598 |
| Pm_1 | 0.363 | 0.125 | 0.004 |
| *Pm_2* | 0.425 | 0.144 | 0.003 |
| Pm_3 | 0.403 | 0.119 | 0.001 |
| Pm_4 | 0.747 | 0.143 | 0.000 |
| Pm_5 | 0.691 | 0.141 | 0.000 |
| Pm_6 | 0.840 | 0.113 | 0.000 |
| Pm_7 | 0.622 | 0.108 | 0.000 |
| Pm_8 | 0.345 | 0.130 | 0.008 |
| Pm_9 | 0.718 | 0.127 | 0.000 |
| Pm_10 | 0.787 | 0.113 | 0.000 |
| Pm_11 | 0.102 | 0.100 | 0.310 |
| Pm_12 | 0.649 | 0.081 | 0.000 |
| Pm_13 | 1.010 | 0.100 | 0.000 |
| week_date |  |  |  |
| 2017w2 | -0.076 | 0.127 | 0.550 |
| *2017w3* | 0.215 | 0.131 | 0.100 |
| *2017w4* | -0.075 | 0.132 | 0.570 |
| 2017w5 | -0.056 | 0.136 | 0.679 |
| 2017w6 | 0.044 | 0.127 | 0.732 |
| 2017w7 | 0.019 | 0.130 | 0.883 |
| 2017w8 | 0.073 | 0.132 | 0.578 |
| 2017w9 | -0.086 | 0.135 | 0.526 |
| 2017w10 | 0.107 | 0.129 | 0.406 |
| 2017w11 | 0.015 | 0.122 | 0.905 |
| 2017w12 | 0.051 | 0.133 | 0.704 |
| 2017w13 | -0.032 | 0.130 | 0.802 |
| 2017w14 | -0.142 | 0.126 | 0.261 |
| 2017w15 | -0.085 | 0.130 | 0.513 |
| 2017w16 | -0.192 | 0.132 | 0.147 |
| 2017w17 | 0.010 | 0.128 | 0.938 |
| 2017w18 | -0.006 | 0.130 | 0.963 |
| 2017w19 | -0.020 | 0.135 | 0.882 |
| 2017w20 | 0.177 | 0.133 | 0.183 |
| 2017w21 | -0.065 | 0.131 | 0.618 |
| 2017w22 | -0.053 | 0.128 | 0.679 |
| 2017w23 | -0.203 | 0.127 | 0.109 |
| 2017w24 | 0.195 | 0.136 | 0.151 |
| 2017w25 | -0.022 | 0.131 | 0.868 |
| 2017w26 | 0.138 | 0.130 | 0.288 |
| 2017w27 | -0.108 | 0.136 | 0.424 |
| 2017w28 | 0.117 | 0.141 | 0.404 |
| 2017w29 | -0.001 | 0.138 | 0.996 |
| 2017w30 | 0.166 | 0.130 | 0.201 |
| 2017w31 | -0.022 | 0.138 | 0.873 |
| 2017w32 | 0.011 | 0.141 | 0.935 |
| 2017w33 | -0.065 | 0.139 | 0.642 |
| 2017w34 | -0.018 | 0.136 | 0.894 |
| 2017w35 | 0.054 | 0.125 | 0.665 |
| 2017w36 | -0.137 | 0.127 | 0.283 |
| 2017w37 | 0.056 | 0.128 | 0.660 |
| 2017w38 | -0.075 | 0.131 | 0.567 |
| 2017w39 | -0.089 | 0.130 | 0.495 |
| 2017w40 | -0.021 | 0.134 | 0.877 |
| 2017w41 | -0.080 | 0.132 | 0.546 |
| 2017w42 | -0.163 | 0.130 | 0.210 |
| 2017w43 | -0.042 | 0.129 | 0.745 |
| 2017w44 | 0.066 | 0.132 | 0.616 |
| 2017w45 | -0.058 | 0.127 | 0.650 |
| 2017w46 | -0.004 | 0.126 | 0.976 |
| 2017w47 | -0.211 | 0.129 | 0.101 |
| 2017w48 | 0.033 | 0.125 | 0.788 |
| 2017w49 | -0.107 | 0.126 | 0.396 |
| 2017w50 | -0.138 | 0.126 | 0.274 |
| 2017w51 | -0.072 | 0.123 | 0.557 |
| 2017w52 | -0.109 | 0.126 | 0.386 |
| _cons | 3.327 | 0.105 | 0.000 |

**Concern#5: Line 325 and 343: I interpreted "instrument" to mean "proxy", an important distinction in my vocabulary. Did you use a true IV approach?**

**Author Response:** Thank you for raising up this detail in the manuscript, maybe it was not enough clear. But in our approach, we used a real IV approach similar to the research (Dogbe and Revoredo-Giha, 2022; J. Banks et al., 1997; Pendakur, 2009; Zhen et al., 2014), which is estimated by replacing the $w_{ht}$ with its mean *(w̅_ht_)*,

$$\bar{y}_{ht}={lnX}_{ht}-\sum_{g=1}^{G} {lnp}_{hgt}\bar{w}_{g}$$

**Concern#6: This is perhaps a thought for the future, but it occurred to me while reading your manuscript. Is there a way to connect your first and second models? To me, you measure first how promotions impact expenditure and then how promotions impact the expenditure share. I believe they may be related. Could equation 5 be a first stage for the EASI model? Perhaps you could isolate the effects of promotional impacts on expenditure share via the impact on expenditure. These are rough thoughts, but I hope they are helpful.**

**Author Response:** Thank you for your insightful suggestion regarding the potential connection between our first and second models. While your idea of using Equation 1 as a first stage for the EASI model to isolate the effects of promotional impacts on expenditure share via the impact on expenditure is intriguing. The first model focuses on quantifying the direct impact of promotions on overall expenditure, to give us a first insight of promotions on household expenditures. While the second model delves into the relationship between promotions and expenditure share of each category and goes beyond the first stage.

The EASI demand system, is already censored in our case, and to solve the problem we had to estimate a probit model, from where we impute an Inverse Mills Ratio (IMR) which is included in the EASI demand as another variable. Additionally, there is the expenditure variable in the EASI model which cause already endogeneity that we instrumented, but if we introduce coefficients from the fixed effect regression, it will cause another endogeneity problem. Therefore, there are some methodological constraints that prevent us from implementing it in this study. While these two aspects are related, attempting to connect them in the suggested manner may introduce complexities that are beyond the scope of our current analysis

However, we appreciate your input and will consider it for future research endeavors. Exploring ways to integrate and align different models to provide a more holistic understanding of the impact of promotions on consumer behavior is certainly a worthwhile endeavor, and we will keep it in mind for future studies.

**References**

Dogbe, W., Revoredo-Giha, C., 2022. Industry levy versus banning promotion on soft drinks in Scotland: A distributional analysis. Food Policy 106, 102191. https://doi.org/10.1016/j.foodpol.2021.102191

Drèze, X., Nisol, P., Vilcassim, N.J., 2004. Do Promotions Increase Store Expenditures? A Descriptive Study of Household Shopping Behavior. Quantitative Marketing and Economics 2, 59–92. https://doi.org/10.1023/b:qmec.0000017035.35940.60

Du, R.Y., Kamakura, W.A., 2008. Where did all that money go? Understanding how consumers allocate their consumption budget. Journal of Marketing 72, 109–131. https://doi.org/10.1509/jmkg.72.6.109

Eguinoa, V.S., 2022. ESTUDIO INFOADEX DE LA INVERSIÓN PUBLICITARIA EN ESPAÑA 2022.

Heien, D., Wessells, C.R., 1990. Demand systems estimation with microdata: A censored regression approach. Journal of Business and Economic Statistics 8, 365–371. https://doi.org/10.1080/07350015.1990.10509807

Hun, M.P., 2011. Practical Guides To Panel Data Modeling : A Step by Step. Public Management and Public Analysis Program 1–53.

Instituto de Estadística de Cataluña [WWW Document], 2024. URL Instituto de Estadística de Cataluña

J. Banks, R. Blundell, A. Lewbel, 1997. Quadratic Engel Curve and Consumer Demand. Review of Economics and Statistics 79, 527–539.

Muth, M.K., Okrent, A.M., Zhen, C., Karns, S.A., 2019. Using scanner data for food policy research., Academic P. ed. Candice Janco.

Naldi, M., Flamini, M., 2014. The CR4 Index and the Interval Estimation of the Herfindahl-Hirschman Index: An Empirical Comparison. SSRN Electronic Journal. https://doi.org/10.2139/ssrn.2448656

Pendakur, K., 2009. EASI made easier. Contributions to Economic Analysis 288, 179–206. https://doi.org/10.1108/S0573-8555(2009)0000288010

Revoredo-Giha, C., Akaichi, F., Leat, P., 2018. Retailers ’ promotions : what role do they play in household food purchases by degree of deprivation ? British Food Journal 04, 14. https://doi.org/https://doi.org/10.1108/BFJ-04-2017-0260

Statista [WWW Document], 2024. URL https://www.statista.com/statistics/535392/grocery-market-share-spain/#:~:text=Grocery market share in Spain 2023&text=Mercadona held the largest share,by Carrefour with 9.7 percent.

Zhen, C., Finkelstein, E.A., Nonnemaker, J.M., Karns, S.A., Todd, J.E., 2014. Predicting the effects of sugar-sweetened beverage taxes on food and beverage demand in a large demand system. American Journal of Agricultural Economics 96, 1–25. https://doi.org/10.1093/ajae/aat049
