# Supplementary material for: Allocation of the household food budget among shopping basket items: How is it influenced by promotions?
Source: PLoS One. 2024 Jun 5;19(6):e0304911. doi: 10.1371/journal.pone.0304911 (PMC11152314; doi:10.1371/journal.pone.0304911)
Supplement: Attachment — Submitted filename: Response to Reviewers.docx [file pone.0304911.s003.docx]

Dear Editor,

Thank you for allowing a resubmission of our manuscript, with an opportunity to address the comments. We would like to thank you for the great interest you have shown in our work, as well as the relevant remarks to improve the article.

Our point-by-point response to the comments are presented below:

**Concern #1: Some in-text citations still do not conform to PLOS ONE’s style. Authors last name should not be written next to numbers.**

**Author response:** Thank you for bringing that to my attention. I ensured that all in-text citations adhere to PLOS ONE’s style guidelines by removing authors' last names next to numbers. I appreciate your feedback.

**Concern #2:** In table 2, please add frequency (N).

**Author response:** Thank you for your feedback. We incorporated the frequency (N) into Table 2 as per your suggestion. This addition will provide readers with a clearer understanding of the sample size for each variable.

**Concern #3:** **Since you followed the two-step procedure suggested by Heien and Wessells (1990), please discus how it differs from Heckman sample selection model and address (Vermeulen, 2001) note in your model case.**

Vermeulen, F. (2001). A note on Heckman-type corrections in models for zero expenditures. *Applied Economics*, *33*(9), 1089–1092. <https://doi.org/10.1080/00036840010004004>

**Author response:** Thank you for your insightful comment. Indeed, the two-step procedure proposed by Heien and Wessells (1990) offers a distinct approach to address sample selection bias compared to the Heckman sample selection model. While both methods aim to mitigate the effects of sample selection bias, they differ in their underlying assumptions and estimation techniques. It has been shown that the Heckman two-step procedure only includes the participating households in the second step of the model. This may cause concerns with the efficiency of the method and the estimated results might only suit the households that participated. The Heien and Wessells two step model is based on the Heckman’s approach but it uses the same households in both of the steps, thus it solves the problem of the Heckman two-step model mentioned above.

According to Heien and Wessells (1990), their approach improved results based on the goodness-of-fit and elasticity values. Even though Wessells' procedure may have inconsistent estimators according to Vermeulen (2001) but the Heckman-selection approach of Heien and Wessels (1990) is supported by Tauchmann (2005) Monte Carlo study of censored demand systems, where he indicates that the approach of Heien and Wessels (1990) has a good performance and might be one of the best choices for practical applications. Additionally, Fernando (2010) mentioned that Heien and Wessells (1990) is one of the two-steps procedures that minimizes computational time. Last but not least, the method of Heien and Wessels (1990) has been widely used by diverse recent works, to cite (Ni Mhurchu et al., 2013), (Sharma et al., 2014), (Caro et al., 2020) and (Gido, 2022).

We made sure to add a note in the manuscript in relation to the method: *even though criticized by (51) but it has been supported by (52) indicating that is one of most suitable methods to account for censoring.* Therefore we added the (Vermeulen, 2001) and (Tauchmann, 2005) references to reference list.

**Concern #4:** **Add the result of the probit model in the appendix section**

**Author response:** Thank you for your suggestion. I included the result of the probit model in the appendix section as per your recommendation.

**Concern #5: I could not find the coefficients value of the IMR and Z (sociodemographic) variables as stated in equation (4). Also, elaborate if you corrected the standard error.**

**Author response:** Thank you for bringing this to our attention. We did not include these coefficients in the results section because we did not report the other coefficients of the equation (4), but we just included the elasticities that we computed form the coefficients of the EASI demand system (equation 4) which are stated in equation 9, 10 and11. That explains why the mentioned coefficients were not included. However, here are the coefficients for you to check them.

| Variable | Eq 1 | Eq 2 | Eq 3 | Eq 4 | Eq 5 | Eq 6 | Eq 7 | Eq 8 | Eq 9 | Eq 10 | Eq 11 | Eq12 |
| --- | --- | --- | --- | --- | --- | --- | --- | --- | --- | --- | --- | --- |
| IMR | 0.07***  (0.009) | 0.012***  (0.004) | 0.094***  (0.003) | 0.09***  (0.008) | 0.039***  (0.008) | 0.117***  (0.006) | 0.070***  (0.008) | 0.124***  (0.005) | 0.029***  (0.009) | 0.146***  (0.006) | 0.098***  (0.003) | 0.002  (0.009) |
| *Household size* |  | | | | | | | | | | | |
| 2 | 0.006  (0.009) | -0.014**  (0.006) | -0.009***  (0.009) | 0.009  (0.009) | -0.027**  (0.013) | -0.000  (0.011) | -0.012  (0.011) | 0.009  (0.007) | 0.011  (0.012) | 0.012  (0.008) | -0.001  (0.005) | -0.014  (0.015) |
| 3 | 0.005  (0.009) | -0.011  (0.006) | -0.005  (0.0004) | 0.013  (0.010) | -0.026*  (0.013) | -0.034***  (0.011) | -0.005  (0.011) | -0.001  (0.007) | 0.005  (0.012) | 0.007  (0.009) | 0.001  (0.005) | 0.017  (0.015) |
| 4 | 0.02*  (0.010) | -0.024***  (0.006) | -0.006  (0.004) | -0.006  (0.010) | -0.035**  (0.014) | -0.041***  (0.012) | 0.025**  (0.011) | -0.010  (0.007) | 0.011  (0.012) | -0.003  (0.009) | 0.012**  (0.005) | 0.002  (0.015) |
| 5 | -0.01  (0.014) | -0.014  (0.008) | -0.0008  (0.006) | 0.006***  (0.01) | -0.059***  (0.019) | -0.062***  (0.016) | -0.015  (0.016) | -0.014  (0.010) | -0.005  (0.0017) | -0.001  (0.012) | -0.002  (0.007) | 0.074***  (0.021) |
| No answer | 0.02  (0.019) | -0.032***  (0.011) | 0.007  (0.008) | -0.001  (0.019) | -0.055**  (0.026) | -0.075***  (0.023) | 0.056**  (0.022) | -0.038***  (0.014) | -0.006  (0.023) | 0.013  (0.017) | 0.004  (0.009) | 0.094***  (0.029) |
| *Social class* |  | | | | | | | | | | | |
| Middle-class | -0.00004  (0.006) | 0.006  (0.004) | -0.009***  (0.003) | -0.006  (0.065) | 0.011**  (0.009) | 0.022***  (0.008) | -0.011  (0.008) | -0.016***  (0.005) | -0.024***  (0.008) | -0.025***  (0.006) | 0.002  (0.003) | -0.002  (0.010) |
| Middle-high class | -0.001  (0.007) | 0.013**  (0.004) | -0.009***  (0.003) | 0.006  (0.007) | 0.010  (0.010) | 0.027***  (0.009) | -0.024***  (0.008) | -0.005  (0.006) | -0.012  (0.009) | -0.023***  (0.006) | -0.0007  (0.004) | -0.007  (0.011) |
| Middle-low class | -0.02**  (0.007) | 0.006  (0.005) | 0.003  (0.003) | -0.010  (0.008) | 0.027**  (0.011) | 0.037***  (0.009) | -0.021**  (0.009) | -0.019***  (0.006) | -0.032***  (0.010) | -0.029***  (0.007) | 0.004  (0.004) | 0.030**  (0.012) |
| *Region* |  | | | | | | | | | | | |
| No metropolitan area | 0.01***  (0.004) | -0.002  (0.003) | -0.004**  (0.002) | -0.0006  (0.005) | -0.014**  (0.006) | 0.013**  (0.005) | 0.0018  (0.005) | 0.011***  (0.003) | 0.019***  (0.006) | -0.005  (0.004) | -0.006***  (0.002) | 0.016**  (0.007) |
| *Age* | -0.0005**  (0.0002) | -0.00007  (0.0001) | -0.0003***  (0.00009) | -0.000006  (0.0002) | 0.00002  (0.0003) | 0.0002**  (0.0002) | -0.0001  (0.001) | 0.0004***  (0.000) | 0.00009  (0.0002) | -0.0004**  (0.0001) | 0.0001  (0.00009) | -0.00005  (0.0003) |

Source: Own elaboration based on Kantar Worldpanel data.

Notes: Bootstrapped standard errors (500 replications) are in parentheses, Sig: Significance level. ***, **, * indicate significance at p <0.001, p < 0.05 and p <0.01, respectively.

**Concern #6:** **Add regression standard error in table 3.**

**Author response:** Thank you for your comment. The regression standard errors have been included in Table 3 of the manuscript already. This query has been addressed within the manuscript.

**Concern #7: Please mention the method used in calculating elasticities' standard error.**

**Author response:** Thank you for your comment. The standard errors for the elasticities were calculated based on bootstrapped standard errors with 500 replications. This method was employed to ensure robustness and reliability in our estimation. Additionally, we added the method under the tables.

References

Caro, J.C., Valizadeh, P., Correa, A., Silva, A., Ng, S.W., 2020. Combined fiscal policies to promote healthier diets: Effects on purchases and consumer welfare. PLoS ONE 15. https://doi.org/10.1371/journal.pone.0226731

Fernando, J., 2010. University of Alberta Three Essays on Canadian Household Consumption of Food Away From Home Department of Rural Economy.

GIDO, E.O., 2022. Household Demand System of African Indigenous Vegetables in Kenya. Review of Agricultural and Applied Economics 25. https://doi.org/10.15414/raae.2022.25.01.94-103

Ni Mhurchu, C., Eyles, H., Schilling, C., Yang, Q., Kaye-Blake, W., Genç, M., Blakely, T., 2013. Food Prices and Consumer Demand: Differences across Income Levels and Ethnic Groups. PLoS ONE 8. https://doi.org/10.1371/journal.pone.0075934

SHARMA, A., HAUCK, K., HOLLINGSWORTH, B., SICILIANI, L., 2014. THE EFFECTS OF TAXING SUGAR‐SWEETENED BEVERAGES ACROSS DIFFERENT INCOME GROUPS. HEALTH ECONOMICS 23, 1159–1184.

Tauchmann, H., 2005. Efficiency of two-step estimators for censored systems of equations: Shonkwiler and Yen reconsidered. Applied Economics 37, 367–374. https://doi.org/10.1080/0003684042000306987

Vermeulen, F., 2001. A note on Heckman-type corrections in models for zero expenditures. Applied Economics 33, 1089–1092. https://doi.org/10.1080/00036840010004004
