# Supplementary material for: Allocation of the household food budget among shopping basket items: How is it influenced by promotions?
Source: PLoS One. 2024 Jun 5;19(6):e0304911. doi: 10.1371/journal.pone.0304911 (PMC11152314; doi:10.1371/journal.pone.0304911)
Supplement: Attachment — Submitted filename: Response to Reviewers.docx [file pone.0304911.s004.docx]

Dear Editor,

Thank you for allowing a resubmission of our manuscript, with an opportunity to address the comments. We would like to thank you for the great interest you have shown in our work, as well as the relevant remarks to improve the article.

Our point-by-point response to the comments are presented below:

**Concern #1: The coefficients of the IMR and other socioeconomics variables were not requested for me to check them as stated in your cover letter, but they were requested to make the results and the methodology of your study transparent and reproducible to other researchers.**

**Author response:** Thank you for highlighting that. We've taken note of your feedback, and we've now included the coefficients of the IMR and other socioeconomic variables in the document for transparency and reproducibility as requested.

| Variable | Eq 1 | Eq 2 | Eq 3 | Eq 4 | Eq 5 | Eq 6 | Eq 7 | Eq 8 | Eq 9 | Eq 10 | Eq 11 | Eq12 |
| --- | --- | --- | --- | --- | --- | --- | --- | --- | --- | --- | --- | --- |
| IMR | 0.067***  (0.009) | 0.124***  (0.004) | 0.094***  (0.003) | 0.090***  (0.008) | 0.040***  (0.008) | 0.117***  (0.006) | 0.071***  (0.008) | 0.124***  (0.005) | 0.029***  (0.009) | 0.146***  (0.006) | 0.098***  (0.003) | 0.003  (0.009) |
| *Household size* |  | | | | | | | | | | | |
| 2 | 0.007  (0.009) | -0.014**  (0.006) | -0.009***  (0.004) | 0.009  (0.009) | -0.027**  (0.013) | -0.000  (0.011) | -0.011  (0.011) | 0.008  (0.007) | 0.011  (0.012) | 0.012  (0.008) | -0.001  (0.005) | -0.014  (0.015) |
| 3 | 0.006  (0.009) | -0.011*  (0.006) | -0.005  (0.0004) | 0.013  (0.010) | -0.026*  (0.013) | -0.034***  (0.011) | -0.004  (0.011) | -0.002  (0.007) | 0.004  (0.012) | 0.007  (0.009) | 0.002  (0.005) | 0.018  (0.015) |
| 4 | 0.017*  (0.010) | -0.024***  (0.006) | -0.006  (0.004) | -0.006  (0.010) | -0.035**  (0.014) | -0.041***  (0.012) | 0.025**  (0.011) | -0.010  (0.007) | 0.011  (0.012) | -0.003  (0.009) | 0.011**  (0.005) | 0.003  (0.015) |
| +5 | -0.002  (0.012) | -0.019**  (0.008) | -0.009  (0.005) | 0.043***  (0.013) | -0.058***  (0.018) | -0.066***  (0.015) | 0.006  (0.015) | -0.021**  (0.010) | -0.005  (0.015) | 0.003  (0.011) | -0.0006  (0.006) | 0.080***  (0.019) |
| *Social class* |  | | | | | | | | | | | |
| Middle-class | -0.001  (0.006) | 0.005  (0.004) | -0.009***  (0.003) | -0.008  (0.007) | 0.020**  (0.009) | 0.021***  (0.008) | -0.009  (0.008) | -0.017***  (0.005) | -0.024***  (0.008) | -0.025***  (0.006) | 0.002  (0.003) | -0.002  (0.010) |
| Middle-high class | -0.001  (0.007) | 0.010**  (0.004) | -0.009***  (0.003) | 0.0004  (0.007) | 0.011  (0.010) | 0.026***  (0.009) | -0.019**  (0.008) | -0.007  (0.005) | -0.012  (0.009) | -0.022***  (0.006) | -0.0002  (0.004) | -0.005  (0.011) |
| Middle-low class | -0.015**  (0.008) | 0.006  (0.005) | 0.004  (0.003) | -0.013  (0.008) | 0.027**  (0.011) | 0.037***  (0.009) | -0.019**  (0.009) | -0.019***  (0.006) | -0.032***  (0.010) | -0.029***  (0.007) | 0.004  (0.004) | 0.031**  (0.012) |
| *Region* |  | | | | | | | | | | | |
| No metropolitan area | 0.013***  (0.004) | -0.003  (0.003) | -0.004**  (0.002) | -0.0005  (0.005) | -0.014**  (0.006) | 0.013**  (0.005) | 0.001  (0.005) | 0.012***  (0.003) | 0.019***  (0.006) | -0.005  (0.004) | -0.007***  (0.002) | -0.016**  (0.007) |
| *Age* | -0.0004**  (0.000) | -0.00009  (0.0001) | -0.0003***  (0.00009) | -0.00008  (0.0002) | 0.00002  (0.0003) | 0.0005**  (0.0002) | -0.0001  (0.0002) | 0.0004***  (0.0001) | 0.00009  (0.0002) | -0.0004**  (0.0001) | 0.0001  (0.00010) | -0.00003  (0.0003) |
| *Residual standard error* | 0.124 | 0.077 | 0.056 | 0.126 | 0.177 | 0.150 | 0.146 | 0.096 | 0.155 | 0.111 | 0.062 | 0.194 |

Source: Own elaboration based on Kantar Worldpanel data.

Notes: Bootstrapped standard errors (500 replications) are in parentheses, Sig: Significance level. ***, **, * indicate significance at p <0.001, p < 0.05 and p <0.01, respectively.

**Concern #2:**

**1. I noticed that in the probit model and the final model you changed the nature of the age variable from categorical variable as reported in table 2 to a numerical variable without informing the readers of such change, which obscures the reproducibility of the paper by other interested researchers.**

**2. The household size variable has 4 respondents who did not answer the question and the authors treated those who did not answer as a regular categorical level, which is incorrect. This is because this category level has few respondents and does not have enough statistical power to compare it and make inference with other categories. Also, the norm when dealing with respondents who do not answer questions is to keep the information as missing and not create a new categorical level. Thus, please re-estimate the model to address this issue.**

**Author response:**

1. Thank you for bringing this to our attention. You're correct that the age variable is categorical (as reported in Table 2) and continuous in the probit model and the final model which may create confusion for the readers. But the age variable present in our dataset is continuous and the age groups created in Table 2 were used just as descriptive of the sample. In any case we removed the age groups created and add the mean of age in Table 2 to avoid any confusion. We apologize for any confusion caused by not explicitly mentioning it in the paper.
2. Thank you for bringing this to our attention. You're absolutely right, and we apologize for the oversight. We acknowledge the importance of handling missing data appropriately and understand that treating non-responses as a separate categorical level may not be statistically sound.

Accordingly, we used the information from previous years of the dataset that we have knowing that households stay in the panel for a period up to three years we checked the households’ size for the four missing households and we found out that they have between more than 5 and 6 members so we added them to the +5 category ( As can be seen in the pictures attached, the one in the right is the dataset that we used and the one in the left is the data we used to replace the missing in our current dataset). We re-estimated the model, taking into account your feedback. The results are presented in the manuscript and here. The difference between the estimations is very tiny and small as you can see in the pictures attached below.We thank you for your constructive criticism, which will undoubtedly improve the quality of our research.

**The results of the old estimation**

- Uncompensated price elasticities
- Promotion elasticities
- Expenditure elasticities

**Results after taking into consideration the missing value issue**

- Uncompensated price elasticities
- Promotion elasticities
- Expenditure elasticities

**Concern #3:** Report the results of residual standard error.

**Author response:** Thank you for your keen observation. The residual standard error has been included in the Table S3 in the appendix as requested.
